# Risk Factors for Hyperglycaemia in Pregnancy in Tamil Nadu, India

**DOI:** 10.1371/journal.pone.0151311

**Published:** 2016-03-18

**Authors:** Karoline Kragelund Nielsen, Peter Damm, Anil Kapur, Vijayam Balaji, Madhuri S. Balaji, Veerasamy Seshiah, Ib C. Bygbjerg

**Affiliations:** 1 Global Health Section, Department of Public Health, University of Copenhagen, Copenhagen, Denmark; 2 World Diabetes Foundation, Gentofte, Denmark; 3 Center for Pregnant Women with Diabetes, Department of Obstetrics, Rigshospitalet, Faculty of Health Sciences, University of Copenhagen, Copenhagen, Denmark; 4 Institute of Clinical Medicine, Faculty of Health and Medical Sciences, University of Copenhagen, Copenhagen, Denmark; 5 Dr. Seshiah Diabetes Research Institute and Dr. Balaji Diabetes Care Centre, Chennai, India; Virgen Macarena University Hospital, School of Medicine, University of Seville, SPAIN

## Abstract

**Introduction:**

Hyperglycaemia in pregnancy (HIP), i.e. gestational diabetes mellitus (GDM) and diabetes in pregnancy (DIP), increases the risk of various short- and long-term adverse outcomes. However, much remains to be understood about the role of different risk factors in development of HIP.

**Objective:**

The aims of this observational study were to examine the role of potential risk factors for HIP, and to investigate whether any single or accumulated risk factor(s) could be used to predict HIP among women attending GDM screening at three centres in urban, semi-urban and rural Tamil Nadu, India.

**Methodology:**

Pregnant women underwent a 75 g oral glucose tolerance test. Data on potential risk factors was collected and analysed using logistical regression analysis. Receiver operating characteristic (ROC) curves, sensitivity, specificity and predictive values were calculated for significant risk factors and a risk factor scoring variable was constructed.

**Results:**

HIP was prevalent in 18.9% of the study population (16.3% GDM; 2.6% DIP). Increasing age and BMI as well as having a mother only or both parents with diabetes were significant independent risk factors for HIP. Among women attending the rural health centre a doubling of income corresponded to an 80% increased risk of HIP (OR 1.80, 95%CI 1.10–2.93; p = 0.019), whereas it was not significantly associated with HIP among women attending the other health centres. The performance of the individual risk factors and the constructed scoring variable differed substantially between the three health centres, but none of them were good enough to discriminate between those with and without HIP.

**Conclusions:**

The findings highlight the importance of socio-economic circumstances and intergenerational risk transmission in the occurrence of HIP as well as the need for universal screening.

## Introduction

Gestational diabetes mellitus (GDM) is one of the most common medical conditions associated with pregnancy and is known to increase the risk of various adverse pregnancy outcomes [1;2]. GDM also increases the risk of future type 2 diabetes in the mother and her offspring [3–6], thereby fuelling the increasing burden of diabetes seen in many countries, including India, where currently 65 million people have diabetes [7]. A number of studies from low and middle income countries have reported relatively high GDM prevalence rates in recent years [8], and studies from high income countries have repeatedly identified pregnant women of Indian/South Asian ethnicity as being at increased risk of GDM [9–11]. A number of studies from India have found GDM prevalence rates ranging from around 5% to 18% [12–20]. Some of these studies have used the Diabetes In Pregnancy Study group India (DIPSI) criteria, a modified version of the World Health Organization (WHO) 1999 criteria relying solely on the two hour 75 g oral glucose tolerance test (OGTT), administered irrespective of last meal time (fasting or non-fasting). Several studies have reported various risk factors associated with higher rates of GDM, mainly focused on traditional risk factors such as age, BMI etc. [12;15–17;21–24], but much remains to be understood particularly about socio-economic factors behind the high rates of GDM.

Studies from high income countries generally find that pregnant women belonging to lower socio-economic groups have a higher risk of developing GDM compared to their counterparts from higher socio-economic strata [25;26]. In India, on the other hand, studies indicate that GDM may be associated with increasing socio-economic status [23], and similar trends have been found for type 2 diabetes [27]. The difference may be explained by the mismatch between the predicted environment for survival programming and the actual environment in adult life due to rapid urbanisation and economic, nutritional and lifestyle transition [28;29]. Individuals pioneering this economic and lifestyle transition are more exposed and therefore vulnerable in the initial phase of epidemiological transition.

Following reports of high GDM prevalence in Tamil Nadu, the state government introduced universal screening for GDM using the DIPSI criteria as part of routine antenatal care [30]. Studying risk factors for GDM in a population where universal testing of all pregnant women is part of public health policy may at first seem superfluous. However, while there are no publicly available data to inform about the extent of coverage and utilisation of the service, we have elsewhere identified a number of barriers to timely GDM screening in Tamil Nadu [31]. Moreover, while most guidelines focus on screening women during the 24 to 28 weeks of gestation, there is concern that some women may manifest hyperglycaemia earlier [32], and late testing, e.g. due to various barriers, may mean missing out on the precious time to take corrective action. The notion of timely detection is important as untreated hyperglycaemia, particularly early during pregnancy, leads to more severe adverse pregnancy outcomes. Implementing multiple testing during pregnancy for all women is not only costly, but operationally challenging. In India, with approximately 27 million births, this means 27 million OGTTs annually to screen all pregnant women at least once during pregnancy; a huge burden to deal with for any health system [33]. A better understanding of the risk factors for GDM may not only add to the knowledge of the pathways leading to GDM, but also inform and enable health care providers to particularly focus on women at high risk for whom earlier and perhaps repeated screening may be of utmost importance.

Moreover, GDM is not the only form of hyperglycaemia which may first be detected during pregnancy. Diabetes mellitus in pregnancy (DIP) is a more severe form of hyperglycaemia in pregnancy (HIP) where diagnostic glucose levels are the same as those used for non-pregnant adults [34]. Data on prevalence of DIP in an Indian setting is lacking and little is known about the characteristics of women with DIP.

Our study was an observational study from Tamil Nadu, India, with a twofold aim: 1) to examine the role of income and education as well as more traditional risk factors for HIP, and 2) to investigate whether any single or accumulated risk factor(s) could be used to predict hyperglycaemic status among women attending GDM screening.

## Methodology

### Design

The data used for this study was collected as part of a large on-going cohort study, designed to identify women with GDM and follow them through pregnancy and postpartum. The cohort includes women attending one of three health centres in Tamil Nadu: 1) an urban private diabetes centre in Chennai catering mainly to the middle class, 2) a semi-urban government maternity health centre in the Saidapet area on the periphery of Chennai, and 3) a rural government primary health centre in the neighbouring Thiruvallur district. For this study we used data from women enrolled in the cohort during its first 25 months–from June 2012 to July 2014. The detailed methods and setup of the cohort have been described previously [35] and here we focus on the methodological aspects and data used in this study.

### Participants and data collection

All pregnant women attending any of the health centres were eligible for inclusion into the cohort unless they had known pre-gestational diabetes. Pregnant women were eligible to participate irrespective of gestational age. The women were briefed about the purpose of the study as well as the screening process, and written signed informed consent was obtained.

A fasting venous blood sample was collected at the time of recruitment. If the women were not fasting they were asked to come back another day fasting. The participants then underwent a 75 g OGTT and venous blood samples were collected two hours later. Participants at the semi-urban and rural centres were asked to come back for a repeat OGTT in the subsequent trimesters if their two hour plasma glucose value was below 140 mg/dl (7.8 mmol/l). If the two hour value was 140 mg/dl (7.8 mmol/l) or more they were diagnosed with GDM and no further OGTTs was performed. At the urban centre the OGTT was not systematically repeated as it was difficult to get the women to drink the glucose solution again, and most often subsequent testing for GDM was in the form of fasting samples and/or postprandial plasma glucose estimation. Only data from the 75 g OGTT is included in this study.

Ethical approval for the study was obtained from the Institutional Ethics Committee at Dr. V. Seshiah Diabetes Research Institute and Dr. Balaji Diabetes Care Centre in Chennai, and permission to carry out the study was granted by the local health authorities.

### Outcome

The main outcome was HIP, but we were also interested in knowing the proportion of pregnant women who would meet the criteria for GDM according to the DIPSI/modified WHO 1999 criteria [36] as well as the criteria for DIP as defined by the WHO 2013 criteria [34]. Therefore, we initially divided the women into three groups: 1) normal glucose tolerance (NGT), 2) GDM defined as two hour plasma glucose value between 140 mg/dl (7.8 mmol/l) and 199 mg/dl (11.0 mmol/l), and 3) DIP defined as fasting plasma glucose of 126 mg/dl (7.0 mmol/l) or more, and/or two hour plasma glucose value of 200 mg/dl (11.1 mmol/l) or more. All blood samples were analysed at the central laboratory of Dr. V. Seshiah Diabetes Research Institute and plasma glucose was estimated by enzymatic glucose oxidase-peroxidase method using the Hitachi fully automated analyser.

### Potential risk factors

Data on potential risk factors was collected by trained field staff. Age was calculated based on the birth date. If this was not known, self-reported age was used. Age was used as a continuous variable in the data analyses. History of diabetes amongst the parents was recorded to construct a categorical variable with four categories: 1) diabetes status in parents not known /no parent has diabetes, 2) only the father has diabetes, 3) only the mother has diabetes, and 4) both parents have diabetes.

Data on gravida, parity, previous abortions, stillbirths, caesarean sections, birth weight of children, and previous diagnosis of polycystic ovarian syndrome (PCOS) was self-reported. A ‘bad obstetric history’ variable was constructed using information on abortions, stillbirths and caesarean sections. A cut-off of 3.5 kg was used to define a macrosomic baby [37;38]. The first day of the last menstrual period was used to calculate gestational age.

Participants were asked to categorise their level of overall physical activity (sedentary/moderate/heavy), and whether they were vegetarians or non-vegetarians.

Highest educational level attained by the woman and her husband were included as two separate categorical variables with the same four categories: 1) primary school or less, 2) secondary school, 3) high school, and 4) college or university. Self-reported monthly household income was recorded and divided by the number of people in the household to obtain per capita monthly income. As the income range was non-normally distributed and quite variable, we transformed the variable using the logarithm to base 2 to take into account that a relative increase in income was more likely to have a bigger impact than an absolute increase.

Pre-pregnancy body mass index (BMI) in kg/m^2^ was calculated using previously measured or self-reported pre-pregnancy weight. If this information was not available, weight noted at the first antenatal care visit was used. Height was recorded in centimetres with the participants standing against the wall in an upright position without footwear using a calibrated stadiometer. Both BMI and height were included as continuous variables in the analyses.

Anaemia was defined according to the WHO recommendations for anaemia in pregnancy as haemoglobin level < 11.0 g/l [39]. For the analyses we used the haemoglobin level measured at the first GDM screening test.

### Data analyses

First, we examined whether the distribution of the various covariates differed significantly between women with NGT, GDM and DIP. For categorical covariates both the overall and the pair-wise comparisons were done using Pearson Chi^2^-test or Fisher’s exact test. For continuous normally distributed covariates the distributions were compared using one-way ANOVA, whereas for non-normally distributed continuous variables Kruskal-Wallis one-way analysis was used for the overall comparison and Mann-Whitney U test for the pair-wise comparisons. To counteract the risk of type I error as a result of the multiple comparisons, the p-values obtained in the pair-wise comparisons were adjusted using the Bonferroni or the Tukey corrections. A critical two-sided p-value of 0.05 or less was considered statistically significant. Covariates were included in the multivariate analyses if the comparisons reached this level after applying the correction.

Logistical regression models were constructed to estimate the effect of the remaining potential risk factors on the outcome variable. For this part of the analysis we combined the GDM and DIP groups into one HIP category. We carried out both bivariate and multivariate analyses to examine the crude and the adjusted associations. Moreover, we tested all unadjusted interactions between income, centre, and education as well as all possible interactions between one of these three and the other covariates by including an interaction term in the models.

Receiver Operating Characteristic (ROC) curves were constructed for the significant continuous variables in the resulting model to identify the cut-off value with the best balance between sensitivity and specificity. The area under the curve was also calculated to assess the risk factors’ ability to discriminate between those with and without HIP. Binary variables were then constructed for all the variables in the resulting model. These were coded with the value 0 for the reference categories/categories that did not significantly increase the risk, and a value of 1 for categories which did increase the risk. A scoring variable was constructed and applied to each of the four settings (overall, rural, semi-urban and urban). ROC curves were computed to identify the most optimum cut-off of the scoring variable. Sensitivity, specificity, positive predictive value and negative predictive value were also calculated for each identified risk factors and number of accumulated risk factors.

Statistical analyses were carried out using SPSS version 20.

## Results

HIP was prevalent in 18.9% (16.3% GDM; 2.6% DIP) of the study population. There was substantial variation in the prevalence between the three centres. At the rural centre 8.0% had GDM and 0.8% had DIP, at the semi-urban centre it was 13.3% and 1.5%, and at the urban centre it was 30.7% and 7.7%.

Table 1 shows the frequency of NGT, GDM and DIP by each of the included covariates. There were significant differences in the mean age and median BMI among women with NGT, GDM and DIP. The three groups also differed in the frequency of anaemia with NGT having the highest frequency and DIP the lowest.

**Table 1:** Distribution of normal glucose tolerance (NGT), gestational diabetes mellitus (GDM), and diabetes in pregnancy (DIP) and association with participant characteristics, n = 4053.

		NGT n = 3287 (81.1%)	GDM n = 659 (16.3%)	DIP n = 107 (2.6%)	P-value overall	P-value NGT vs GDM	P-value NGT vs DIP	P-value GDM vs DIP
Health centre (n = 4053)	*Govt*. *rural (n = 373)*	340 (91.2%)	30 (8.0%)	3 (0.8%)	<0.001*	<0.001*** **	<0.001*** **	0.003**** **
	*Govt*. *semi-urban (n = 2884)*	2456 (85.2%)	385 (13.3%)	43 (1.5%)				
	*Private urban (n = 796)*	491 (61.7%)	244 (30.7%)	61 (7.6%)				
Gestational age at first test (weeks) (n = 4053)	*Median (Q1/Q3)*	21 (17/24)	20 (15/24)	16 (10/27)	**<0.001**^a^	**<0.001**^b^	**<0.001**^b^	0.297^b^
Gestational age at last test/diagnosis (weeks) (n = 4053)	*Median (Q1/Q3)*	26 (20/32)	21 (15/27)	18 (10/27)	**<0.001**^a^	**<0.001**^b^	**<0.001**^b^	0.108^b^
Age (years) (n = 4046)	*Means (SD±)*	24.2 (3.9)	26.8 (4.5)	28.1 (4.4)	**<0.001**^c^	**<0.001**^c^	**<0.001**^c^	**0.005**^c^
Height (cm) (n = 3537)	*Means (SD±)*	154.2 (6.1)	155.3 (6.2)	155.6 (6.8)	**<0.001**^c^	**<0.001**^c^	0.079^c^	0.878^c^
BMI (kg/m^2^) (n = 3410)	*Median (Q1/Q3)*	21.6 (18.8/24.9)	24.1 (20.9/27.8)	26.7 (23.3/28.9)	**<0.001**^a^	**<0.001**^b^	**<0.001**^b^	**<0.001**^b^
Monthly income per household member (Indian rupees) (n = 3863)	*Median (Q1/Q3)*	2000 (1333/3333)	2500 (1666/5416)	2500 (1666/6583)	**<0.001**^a^	**<0.001**^b^	**0.003**^b^	1.000^b^
Education (n = 3950)	*Primary school or less (n = 454)*	393 (86.6%)	53 (11.7%)	8 (1.7%)	<0.001*	<0.001*	0.006*	1.000*
	*Secondary school (n = 1827)*	1551 (84.9%)	238 (13.0%)	38 (2.1%)				
	*High school (n = 580)*	490 (84.5%)	78 (13.4%)	12 (2.1%)				
	*College or university* (n = 1089)	787 (72.3%)	261 (24.0%)	41 (3.7%)				
Husband’s education (n = 3947)	*Primary school or less (n = 506)*	444 (87.7%)	54 (10.7%)	8 (1.6%)	<0.001*	<0.001*	<0.001*	1.000*
	*Secondary school (n = 2008)*	1714 (85.4%)	255 (12.7%)	39 (1.9%)				
	*High school (n = 402)*	327 (81.3%)	65 (16.2%)	10 (2.5%)				
	*College or university (n = 1031)*	732 (71.0%)	257 (24.9%)	42 (4.1%)				
Type of family (n = 3953)	*Nuclear (n = 1983)*	1628 (82.1%)	301 (15.2%)	54 (2.7%)	0.834*	-	-	-
	*Joint (n = 1970)*	1594 (80.9%)	331 (16.8%)	45 (2.3%)				
Overall physical activity level (n = 3955)	*Sedentary (n = 840)*	725 (86.3%)	105 (12.5%)	10 (1.2%)	<0.001**	0.003**	0.012**	0.186**
	*Moderate (n = 3096)*	2481 (80.1%)	526 (17.0%)	89 (2.9%)				
	*Heavy (n = 19)*	17 (89.4%)	1 (5.3%)	1 (5.3%)				
Dietary habits (n = 4032)	*Vegetarian (n = 180)*	154 (85.6%)	24 (13.3%)	2 (1.1%)	0.834**	-	-	-
	*Non-vegetarian (n = 3852)*	3119 (81.0%)	630 (16.3%)	103 (2.7%)				
Parents with diabetes (n = 4013)	*Not known/none of the parents have diabetes (n = 3204)*	2713 (84.7%)	434 (13.5%)	57 (1.8%)	<0.001*	<0.001*	<0.001**	0.345*
	*Father only (n = 423)*	305 (72.1%)	96(22.7%)	22 (5.2%)				
	*Mother only (n = 271)*	183 (67.5%)	72 (26.6%)	16 (5.9%)				
	*Both (n = 115)*	58 (50.4%)	48 (41.7%)	9 (7.8%)				
Previous diagnosis of GDM (n = 3774)	*No (n = 3731)*	3055 (81.9%)	588 (15.8%)	88 (2.3%)	<0.001 **	0.222*	<0.001**	0.042**
	*Yes (n = 43)*	27 (62.8%)	10 (23.2%)	6 (14.0%)				
Diagnosis of PCOS (n = 4030)	*No (n = 3933)*	3203 (81.4%)	629 (16.0%)	101 (2.6%)	0.048**	0.021*	0.855**	1.000**
	*Yes (n = 97)*	68 (70.1%)	25 (25.8%)	4 (4.1%)				
Parity (n = 4007)	*0 (n = 2176)*	1765 (81.1%)	363 (16.7%)	48 (2.2%)	0.549**	-	-	-
	*1 (n = 1686)*	1375 (81.5%)	256 (15.2%)	55 (3.3%)				
	≥ *2 (n = 145)*	114 (78.6%)	27 (18.6%)	4 (2.8%)				
Gravida (n = 4047)	*1 (n = 1996)*	1640 (82.2%)	309 (15.5%)	47 (2.3%)	0.084*** **	-	-	-
	*2 (n = 1538)*	1249 (81.2%)	251 (16.3%)	38 (2.5%)				
	≥ *3 (n = 513)*	393 (76.6%)	98 (19.1%)	22 (4.3%)				
Previous abortion (n = 4043)	*No (n = 3468)*	2855 (82.3%)	528 (15.2%)	85 (2.5%)	<0.001*	<0.001*	0.066*	1.000*
	*Yes (n = 575)*	424 (73.8%)	129 (22.4%)	22 (3.8%)				
Previous stillbirth (n = 4053)	*No (n = 3958)*	3211 (81.1%)	641 (16.2%)	106 (2.7%)	1.000**	-	-	-
	*Yes (n = 95)*	76 (80.0%)	18 (18.9%)	1 (1.1%)				
Previous caesarean section (n = 3915)	*No (n = 3248)*	2670 (82.2%)	509 (15.7%)	69 (2.1%)	<0.001*	0.027*	<0.001*	0.105*
	*Yes (n = 667)*	509 (76.3%)	129 (19.3%)	29 (4.3%)				
Previous given birth to a baby weighing 3,5 kg or more (n = 3894)	*No (n = 3632)*	2959 (81.5%)	592 (16.3%)	81 (2.2%)	<0.001*	0.972*	<0.001*	<0.001*
	*Yes (n = 264)*	199 (75.4%)	47 (17.8%)	18 (6.8%)				
Bad obstetric history (abortion, stillbirth, or caesarean section) (n = 3941)	*No (n = 2776)*	2317 (83.5%)	402 (14.5%)	57 (2.1%)	<0.001*	<0.001*	0.006*	1.000*
	*Yes (n = 1165)*	882 (75.7%)	242 (20.8%)	41 (3.5%)				
Anaemia at first test (n = 3771)	*No (n = 2465)*	1994 (80.9%)	401 (16.3%)	70 (2.8%)	<0.001*	<0.001*** **	<0.001*** **	0.042*** **
	*Yes (n = 1306)*	1133 (86.7%)	160 (12.3%)	13 (1.0%)				

Listed p values are Bonferroni or Tukey adjusted.

* Chi^2^ test,

** Fisher’s exact test,

^a^ Kruskal-Wallis one-way analysis of variance,

^b^ Mann-Whitney U test,

^c^ One-way ANOVA with multi-comparisons adjusted using Tukey post hoc test.

Compared to women with NGT, women with GDM and DIP had higher per capita monthly income as well as higher educational attainment among themselves and their husbands. No statistically significant difference was noted between the GDM and DIP groups. Distribution of self-reported physical activity, parental history of diabetes, and the combined ‘bad obstetric history’ variable likewise differed between the NGT and the two HIP groups, but not between the GDM and the DIP groups. In contrast, the distribution of previous diagnosis of GDM and previously given birth to a macrosomic baby was significantly different between the DIP group and the GDM and NGT groups, but not between the GDM and the NGT groups. There was no statistically significant difference between the groups in terms of type of family, dietary habits, parity, gravida and stillbirths. Since the risk factor profiles for the GDM and DIP groups were very similar they were combined into one HIP group for the logistic regression analyses.

In the bivariate logistical regression analyses (Table 2) the following significantly increased the risk of HIP: attending the urban or semi-urban health centre, advancing age, increasing height, BMI, income, and educational status of both self and husband, moderate physical activity level, having one or both parents with diabetes, having ‘bad obstetric history’, given birth to a large baby and diagnosis of PCOS. Anaemia in turn reduced the risk of HIP.

**Table 2:** Bivariate and multivariate analyses of association between potential risk factors and hyperglycaemia in pregnancy (HIP).

		OR crude [95%CI]	p-value	OR adjusted model 1 [95%CI]	p-value	OR adjusted model 2 [95%CI]	p-value	OR adjusted model 3 [95%CI]	p-value	OR adjusted model 4 [95%CI]	p-value
Health centre	*Govt*. *rural*	REF		REF		REF		REF		REF	
	*Govt*. *semi-urban*	1.80 [1.24–2.60]	**0.002**	1.47 [1.00–2.16]	**0.047**	1.53 [1.04–2.27]	**0.033**	1.50 [1.01–2.23]	**0.047**	1.50 [1.01–2.23]	**0.047**
	*Private urban*	6.40 [4.36–9.40]	**<0.001**	2.65 [1.75–4.01]	**<0.001**	2.69 [1.63–4.46]	**<0.001**	2.64 [1.58–4.40]	**<0.001**	2.64 [1.58–4.40]	**<0.001**
Age	*Year*	1.16 [1.14–1.19]	**<0.001**	1.13 [1.11–1.16]	**<0.001**	1.13 [1.10–1.15]	**<0.001**	1.12 [1.10–1.15]	**<0.001**	1.11 [1.09–1.14]	**<0.001**
Height	*Cm*	1.03 [1.02–1.04]	**<0.001**	1.01 [1.00–1.03]	0.071	1.01 [0.99–1.02]	0.517	1.00 [0.99–1.02]	0.620	1.00 [0.98–1.02]	0.816
BMI	*Kg/m*^*2*^	1.12 [1.10–1.14]	**<0.001**	1.08 [1.05–1.10]	**<0.001**	1.07 [1.05–1.10]	**<0.001**	1.07 [1.05–1.09]	**<0.001**	1.06 [1.04–1.09]	**<0.001**
Log2 monthly income per household member	*Doubling in income*	1.32 [1.24–1.40]	**<0.001**	1.09 [1.02–1.17]	**0.017**	1.05 [0.96–1.14]	0.294	1.05 [0.97–1.14]	0.252	0.96 [0.87–1.06]	0.365
Education	*Primary school or less*	REF		REF		REF		REF		REF	
	*Secondary school*	1.15 [0.85–1.55]	0.370	1.20 [0.85–1.69]	0.311	1.18 [0.84–1.68]	0.341	1.16 [0.82–1.65]	0.405	1.17 [0.82–1.67]	0.377
	*High school*	1.18 [0.83–1.68]	0.347	1.21 [0.80–1.81]	0.366	1.23 [0.82–1.84]	0.324	1.19 [0.79–1.80]	0.405	1.19 [0.79–1.80]	0.417
	*College or university*	2.47 [1.83–3.34]	**<0.001**	1.67 [1.18–2.38]	**0.004**	1.46 [1.00–2.14]	0.050	1.39 [0.94–2.04]	0.096	1.20 [0.81–1.79]	0.367
Husband’s education	*Primary school or less*	REF		REF		REF		REF		REF	
	*Secondary school*	1.23 [0.92–1.65]	0.169	1.11 [0.80–1.55]	0.534	1.09 [0.77–1.54]	0.619	1.12 [0.79–1.59]	0.522	1.12 [0.79–1.59]	0.525
	*High school*	1.64 [1.14–2.37]	**0.008**	1.41 [0.94–2.13]	0.100	1.34 [0.87–2.06]	0.189	1.35 [0.87–2.09]	0.184	1.34 [0.87–2.08]	0.188
	*College or university*	2.93 [2.17–3.94]	**<0.001**	1.71 [1.22–2.41]	**0.002**	1.41 [0.93–2.12]	0.102	1.43 [0.94–2.17]	0.092	1.29 [0.84–1.98]	0.240
Overall physical activity level	*Sedentary*	REF		REF		REF		REF		REF	
	*Moderate*	1.56 [1.26–1.94]	**<0.001**	1.24 [0.97–1.58]	0.084	1.10 [0.86–1.42]	0.447	1.11 [0.86–1.43]	0.426	1.08 [0.84–1.40]	0.539
	*Heavy*	0.74 [0.17–3.25]	0.692	0.66 [0.14–3.02]	0.588	0.67 [0.15–3.11]	0.610	0.67 [0.15–3.10]	0.609	0.67 [0.14–3.07]	0.601
Parents with diabetes	*Not known /none have*	REF		REF		REF		REF		REF	
	*Father only*	2.14 [1.69–2.70]	**<0.001**	1.48 [1.14–1.92]	**0.004**	1.24 [0.93–1.65]	0.151	1.25 [0.93–1.67]	0.140	1.15 [0.86–1.55]	0.347
	*Mother only*	2.66 [2.03–3.49]	**<0.001**	1.79 [1.31–2.44]	**<0.001**	1.69 [1.21–2.35]	**0.002**	1.76 [1.26–2.45]	**0.001**	1.63 [1.17–2.28]	**0.004**
	*Both*	5.43 [3.72–7.92]	**<0.001**	3.21 [2.14–4.80]	**<0.001**	2.88 [1.86–4.48]	**<0.001**	2.85 [1.83–4.45]	**<0.001**	2.46 [1.56–3.88]	**<0.001**
Previous diagnosis of GDM	*No*	REF		REF		REF		REF		REF	
	*Yes*	2.68 [1.44–4.99]	**0.002**	2.13 [1.04–4.34]	**0.039**	2.19 [1.05–4.56]	**0.037**	1.93 [0.91–4.11]	0.088	2.09 [0.99–4.44]	0.055
Diagnosis of PCOS	*No*	REF		REF		REF		REF		REF	
	*Yes*	1.87 [1.20–2.91]	**0.005**	1.29 [0.76–2.19]	0.340	1.21 [0.69–2.13]	0.505	1.19 [0.66–2.12]	0.566	1.11 [0.62–1.99]	0.735
Diagnosis of PCOS	*No*	REF		REF		REF		REF		REF	
	*Yes*	1.87 [1.20–2.91]	**0.005**	1.29 [0.76–2.19]	0.340	1.21 [0.69–2.13]	0.505	1.19 [0.66–2.12]	0.566	1.11 [0.62–1.99]	0.735
Previous caesarean section	*No*	REF		REF		REF				REF	
	*Yes*	1.43 [1.17–1.75]	**<0.001**	0.91 [0.72–1.15]	0.432	1.00 [0.78–1.28]	0.991	-	-	1.00** [0.78–1.28]	0.989
Previous baby weighing 3.5 kg or more	*No*	REF		REF		REF		REF		REF	
	*Yes*	1.44 [1.07-1-92]	**0.015**	1.09 [0.78–1.51]	0.628	1.09 [0.77–1.55]	0.612	1.04 [0.73–1.48]	0.836	1.04 [0.73–1.48]	0.845
Bad obstetric history (abortion, stillbirth, or caesarean section)	*No*	REF		REF		REF		REF		REF	
	*Yes*	1.62 [1.37–1.91]	**<0.001**	1.14 [0.94–1.39]	0.192	1.20 [0.98–1.48]	0.086	1.20 [0.98–1.48]	0.086	1.18 [0.96–1.45]	0.121
Anaemia at first test	*No*	REF		REF		REF		REF		REF	
	*Yes*	0.65 [0.54–0.78]	**<0.001**	0.79 [0.64–0.99]	**0.038**	0.81 [0.65–1.02]	0.068	0.80 [0.64–1.009	0.052	0.80 [0.64–1.00]	0.052
Log2 monthly income per household member * centre	*Overall*		**0.022**		0.071		0.073		**0.030**		
	*Doubling in income at rural centre*	1.70 [1.07–2.71]	**0.024**	1.61 [1.00–2.60]	**0.049**	1.60 [0.99–2.58]	0.054	1.80 [1.10–2.93]	**0.019**	-	**-**
	*Doubling in income at semi-urban centre*	1.01 [0.90–1.14]	0.840	0.98 [0.85–1.14]	0.801	0.96 [0.83–1.12]	0.629	0.96 [0.83–1.12]	0.604	-	**-**
	*Doubling in income at urban centre*	0.90 [0.80–1.02]	0.085	0.91 [0.80–1.04]	0.152	0.90 [0.79–1.03]	0.127	0.91 [0.80–1.04]	0.153	-	**-**

Model 1 adjusted for age, parental diabetes and BMI; Model 2: model 1 + education and income; Model 3: model 2 + bad obstetric history; Model 4: model 3 + centre

** Bad obstetric history not included in model 4.

In adjusted analyses (Table 2), some of the associations were attenuated, and only health centre, age, BMI, and having only a mother or both parents with diabetes remained statistically significant. The interaction term ‘log2income and centre’ also remained significant after adjustment, showing that among women attending the rural health centre a doubling in household income corresponded to a 80% increased risk for HIP (OR 1.80, 95% CI 1.10–2.93; p = 0.019). Among women attending the semi-urban and urban centres there was no statistically significant association between income and HIP. Previous GDM diagnosis, history of abortion and presence of anaemia were significant in some models, but not in all models.

Three variables–age, BMI and having only a mother or both parents with diabetes–were found to be significant risk factors for HIP (Table 2) which remained unaffected by adjustments and effect modification. The ROC curves for age and BMI showed that the overall cut-off values yielding the best balance between sensitivity and specificity were 25 years for age (69.3% sensitivity and 57.7% specificity) and 22.3kg/m^2^ for BMI (66.6% sensitivity and 57.4% specificity).

We used these cut-off values to compute the scoring variable. Thus, it was possible to have between zero and three risk factors.

**Fig 1:**
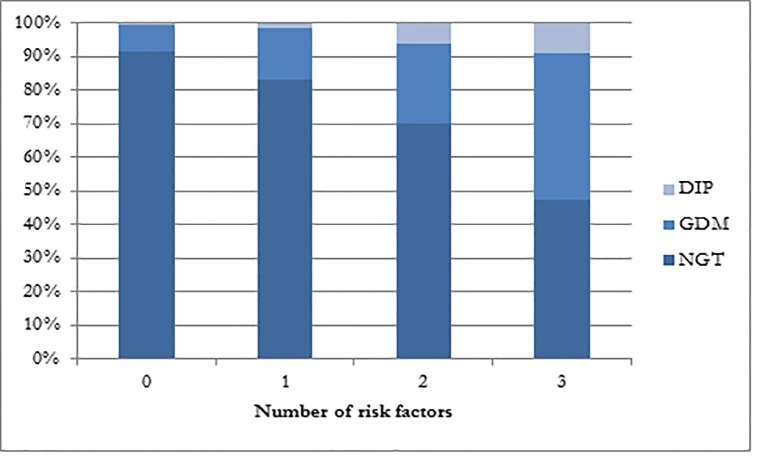
Distribution of risk factors according to hyperglycaemic status among pregnant women, n = 3377^†^. ^†^ Only subjects with data on all three risk factors included.

Fig 1 shows the distribution of number of risk factors among the three groups. Among women with none of the three risk factors 91.7% were in the NGT group, 7.9% had GDM and only 0.4% met the diagnostic criteria for DIP. In contrast, more than half of women with all three risk factors had HIP (GDM 43.5% and DIP 9.2%).

The sensitivity, specificity, and positive and negative predictive values for each risk factor and number of risk factors as applied to the whole group are presented in Table 3. In Table 4 they are shown as applied to the three health centres separately. Overall, age ≥ 25 years and BMI ≥ 22.3 correctly identified two thirds of women with HIP, but there were substantial differences between the centres. Both risk factors had a specificity of around 57%.

**Table 3:** Sensitivity, specificity, positive predictive values and negative predictive values for individual and accumulated number of risk factors and hyperglycaemia in pregnancy (HIP). Based on those with data for all three risk factors, n = 3377.

	Total number with risk factor (%)	Sensitivity	Specificity	Positive predictive value	Negative predictive value
Age 25 years or more	1606 (47.6%)	69.3%	57.7%	28.3%	88.7%
BMI 22.3 or more	1595(47.2%)	66.6%	57.4%	27.3%	87.7%
Mother only or both parents with diabetes	349 (10.3%)	20.9%	92.2%	39.3%	82.9%
At least 1 risk factor	2275 (67.4%)	86.1%	37.1%	24.8%	91.7%
At least 2 risk factors	1091 (32.3%)	55.9%	73.4%	33.5%	87.4%
3 risk factors	184 (5.4%)	14.8%	96.8%	52.7%	82.5%

**Table 4:** Sensitivity, specificity, positive predictive values and negative predictive values for individual and accumulated number of risk factors and hyperglycaemia in pregnancy (HIP) stratified according to health centre. Based on those with data for all three risk factors, n = 3377.

	Rural centre				Semi-urban centre				Urban centre			
	Sensitivity	Specificity	Positive predictive value	Negative predictive value	Sensitivity	Specificity	Positive predictive value	Negative predictive value	Sensitivity	Specificity	Positive predictive value	Negative predictive value
Age 25 years or more	42.4%	74.3%	14.0%	92.9%	62.2%	62.1%	21.9%	90.6%	80.3%	28.2%	41.0%	69.6%
BMI 22.3 or more	51.5%	66.2%	13.1%	93.2%	53.4%	62.8%	19.7%	88.7%	83.0%	29.2%	42.2%	73.4%
Mother or both parents with diabetes	0.0%	98.2%	0.0%	90.8%	12.2%	95.1%	29.9%	86.4%	33.0%	76.9%	47.1%	64.8%
At least 1 risk factor	69.7%	51.2%	12.4%	94.5%	78.4%	41.5%	18.7%	91.8%	96.6%	9.3%	39.9%	81.5%
At least 2 risk factors	24.2%	87.1%	15.7%	92.1%	41.5%	79.6%	25.9%	88.8%	75.5%	38.1%	43.2%	71.4%
3 risk factors	0.0%	99.4%	0.0%	91.0%	7.9%	98.8%	53.1%	86.2%	24.1%	86.9%	53.3%	64.8%

Having a mother only or both parents with diabetes had low sensitivity but high specificity and positive predictive value. Overall, the predictive values differed considerably between the centres with particularly low positive predictive values at the rural centre. The highest positive predictive value (47.1%) was having a mother only or both parents with diabetes at the urban centre.

Using number of risk factors to identify HIP also resulted in low positive predictive values at the rural centre (0.0%-15.7%). At the semi-urban and urban centres using a cut-off of 3 risk factors yielded a positive predictive value of around 53%. However, it identified less than 8% of cases at the semi-urban centre and only about a quarter at the urban centre. Overall, having any one risk factor yielded the highest sensitivity (86.1%), with a specificity of 37.1%. The ROC curve for the scoring variable identified a cut-off value of two or more risk factors as giving the best possible balance when combining all three centres (55.9% sensitivity and 73.4% specificity). However, at the rural centre a better balance was found using one risk factor as cut-off. The area under the curve for the ROC was 0.688 (95% CI 0.665–0.711, p<0.001) for the centres combined, and it was even lower for the individual centres, suggesting that the scoring variable did not discriminate very well between women with and without HIP.

## Discussion

In this observational study from Tamil Nadu we investigated the occurrence of HIP and potential risk factors associated with it. We further investigated whether these could be used to guide the screening process by predicting hyperglycaemic status. The study confirmed that GDM is highly prevalent in this population as found in earlier studies [12;13;15–17]. The study also showed that DIP, the more severe form of HIP, is also present among pregnant women even in these settings. The pregnant women attending the private urban centre had been referred to the centre, and the high prevalence rates of both GDM and DIP found in this centre may be due to selection bias. On the other hand, only the result of the OGTT at their first visit was included, and some of them may have developed GDM later in their pregnancy. Therefore, the prevalence rate of GDM may also be underestimated. In the semi-urban and rural centres, women were screened as part of the antenatal care programme, and testing was also repeated if they attended later in pregnancy. These results may, thus, be more representative of the actual burden of HIP. Moreover, partial data was missing for 42.3% of the women at the urban centre, and analyses of these women (not presented here) showed that they tended to have higher rates of HIP, were older, had higher BMI and income compared to women without partial missing data. This has implications for the multivariate analyses, where associations may be underestimated, and for the findings in the sensitivity and specificity analysis. We, therefore, also calculated the sensitivity and specificity estimations from worst and best case scenarios (data not shown) where missing data was coded as having the risk factor (worst case) or not (best case). Doing so, either did not markedly change or resulted in lowering the sensitivity/specificity except for family history of diabetes where the performance was slightly improved.

In the final analyses, after adjusting for potential confounders, we, not unexpectedly, found that advancing age and BMI, and having a mother only or both parents with diabetes increased the risk of HIP. Furthermore, a doubling of income increased the risk amongst women attending the rural centre, whereas a doubling of income had no significant effect for women attending the semi-urban or urban centres.

Age, BMI and family history of diabetes have been identified as risk factors in a number of other studies from India [12;15–17;21–24]. Studies have also identified previous history of macrosomia, GDM and abortions as risk factors [15;18;21;23;24]. However, these were not significant risk factors in our adjusted models, though previous abortion was borderline significant after adjustment for health centre. This may be explained by a higher frequency of past abortion among women attending the urban centre. Previous history of GDM was significant until adjustment for bad obstetric history was included. However, ‘bad obstetric history’ may be a marker of previous undiagnosed GDM and so this adjustment may be considered an overcorrection.

It is estimated that almost 32 million people with diabetes in India are undiagnosed [7]. Therefore, it is possible that the reason for the fairly low sensitivity of family history of diabetes is due to low awareness and testing rates for diabetes. The low sensitivity in the rural area could also be a reflection of a ‘more recent’ economic transition in the life of rural women with HIP. Results could also be biased if certain groups are less or more likely to have been tested. For instance, if the women’s mothers were more likely to be tested than the fathers, or if testing is linked to certain socioeconomic groups as found in high income settings [40;41]. Available data, however, suggests that the proportion of undiagnosed diabetes is higher or the same in women in India compared to men [42]. Similar bias could have occurred for data on GDM in previous pregnancies as well as PCOS diagnosis. Data on whether the women were tested for PCOS or GDM in previous pregnancies was not available and could not be further elicited. In our study, the estimation of the women’s weight and, thus, BMI may also be prone to some bias as it is in part based on self-reported weight or weight measured at first antenatal care visit. The latter may have led to an overestimation of BMI, whereas the opposite could be the case for self-reported weight. A similar situation also pertains to the use of self-reported physical activity.

Recent years have seen an increased focus on the intergenerational transmission of risks for non-communicable diseases through developmental programming or epigenetic changes in relation to both GDM and type 2 diabetes. A central focus has been the mismatch hypothesis where changes in living conditions and social determinants from life in-utero till adulthood fuel the risk of hyperglycaemia [43]. Consequently, inclusion of socio-economic risk factors and how these interact with clinical risk factors, as we have done in this study, may help understand the pattern of risk factors for HIP over time, place and generations and hopefully help identify vulnerable populations.

The association of lower BMI, lower income, lower education, higher level of physical activity, higher rates of anaemia and higher attendance at the government health centres in the NGT group noted in our study clearly indicates difficult life conditions, relatively unaffected by economic transition and, thus, protected from metabolic derangements. A relatively higher income and slightly improved living condition in this group, who were programmed to survive in much less, may trigger metabolic aberrations particularly during pregnancy. This is further corroborated by the fact that among women attending the rural health centre a doubling in income caused an 80% increased risk of HIP. In a study from Tamil Nadu, Ramachandran et al. [29] showed a threefold increased prevalence of diabetes over a fourteen year period in a rural population transitioning from poverty to subsistence living. In our study, which took place 10 years after Ramachandran’s, there was no significant effect of income among women attending the semi-urban and urban centres. The centres may therefore represent different stages of the economic transition. Stuckler investigated the effect of macrosocial and macroeconomic forces on chronic disease mortality and showed that increasing per capita income in low income countries was associated with greater mortality rates of non-communicable diseases like diabetes, whereas it was associated with lesser rise or reduced rates in middle income countries, and reduced rates in high income countries. Stuckler concluded that prevailing demographic explanations such as population aging are insufficient on methodological, empirical and policy grounds [44]. Our study suggests similar trends and conclusions for HIP morbidity but at a meso-level. Importantly however, BMI, anaemia and socio-economic status (and its relative change) are closely linked to lifestyle, especially diet [45]. In our study, very limited data was available on diet as well as physical activity and future studies should seek to capture these in greater detail.

Despite a 42% higher rate of paternal diabetes (538 fathers vs 386 mothers with positive history) in the study population, the highly significant association of HIP with maternal diabetes in our study is an important finding. Only one case-control study from India has previously reported similar findings but this was unadjusted for potential confounders [21]. It cannot be ruled out that this finding is due to reporting bias or greater similarities in lifestyle behaviours among mothers-daughters than fathers-daughters. However, given that women with GDM have a seven to eight fold higher risk of type 2 diabetes [46], it is not farfetched to conjecture that many women with HIP in our study, particularly in the urban and semi-urban centres, may have been themselves offspring of HIP mothers not identified at that time due to lack of facilities, awareness or resources. Focusing on offspring exposed to hyperglycaemia in utero for special preventive action is of course important, but in the context of HIP, identifying girls born to HIP mothers may be even more relevant not only for future prevention of type 2 diabetes, but also to ensure targeted early testing for HIP, when they themselves become pregnant.

In the second part of the study, we investigated whether the identified risk factors could be used to predict or serve as a pre-screening tool for identifying pregnant women at risk of HIP. None of the single or accumulated risk factors had more than a modest balance between sensitivity, specificity and positive predictive value, and there were substantial differences in these balances between the health centres.

Overall, the measure yielding the highest sensitivity was having one risk factor. Thus, by screening only women with one or more risk factors, 86.1% of all the HIP cases would be identified while omitting the need for 32.6% of OGTTs. However, when applied to the government centres it would miss 20–30% of HIP cases, and can therefore not be recommended. This study therefore supports the current Tamil Nadu state policy of screening all pregnant women for HIP.

However, given the challenges in completing GDM screening in a timely manner [31], the positive predictive values identified in this study can guide health care providers to identify women, who should receive special attention and assistance through the screening process. This applies particularly to women with one or more risk factors and especially women with a mother or both parents with diabetes.

## Conclusion

The present study confirms previous reports of high GDM prevalence rates in Tamil Nadu and provides new data on the occurrence of DIP. Advancing age and BMI are important risk factors, but their sensitivity and positive predictive values differed substantially between the rural, semi-urban and urban settings indicating differences in the risk characteristics of the populations living there. This was further supported by the finding that a doubling of income was associated with increasing risk for HIP among women at the rural centre, but not among women at the two other centres. Together with the finding that maternal but not paternal diabetes alone increased the risk, these results highlight the importance of socio-economic status and potential intergenerational impact on HIP. Our attempt to construct a risk factor based scoring variable to guide screening efforts provided some indication, but none of the risk factors or their accumulation were robust enough to clearly discriminate between those with and without HIP in all the settings, underlining the need for universal screening for GDM/DIP in Tamil Nadu.

## Supporting Information

S1 Supporting Information Data set. (SAV)
